# Discriminating Value of Calprotectin in Disease Activity and Progression of Nonradiographic Axial Spondyloarthritis and Ankylosing Spondylitis

**DOI:** 10.1155/2017/7574147

**Published:** 2017-05-24

**Authors:** Jinxian Huang, Zhihua Yin, Guoxiang Song, Shengjin Cui, Jinzhao Jiang, Lijun Zhang

**Affiliations:** ^1^Rheumatology Department, The University of Hong Kong-Shenzhen Hospital, Shenzhen, China; ^2^Rheumatology Department, The Fourth People's Hospital of Shenzhen, Shenzhen, China; ^3^The Third People's Hospital of Shenzhen, Shenzhen, China

## Abstract

It has been controversial whether ankylosing spondylitis (AS) and nonradiographic axial spondyloarthritis (nr-axSpA) are separate or different phases of radiographic progression. We determined that serum calprotectin level (ng/ml) was higher in AS (15.30 ± 6.49) and nr-axSpA (17.76 ± 8.59) patients than in healthy individuals (7.40 ± 2.67). No difference was observed in calprotectin level between these two groups. Elevated calprotectin was positively correlated with ESR, CRP, BASDAI, and ASDAS as well as SPARCC scoring and had no correlation with BASFI and mSASSS. No correlation was observed between calprotectin and Wnt/*β*-catenin pathway markers. Serum calprotectin can be used as a marker for inflammation in both nr-axSpA and AS, while it does not contribute to the discrimination of AS and nr-axSpA. Calprotectin-mediated inflammation was not correlated with principle effectors of Wnt/*β*-catenin pathway, indicating that inflammation and bone fusion might be separate processes of the disease.

## 1. Introduction

Spondyloarthritis (SpA) contains a large group of chronic inflammatory rheumatic disorders characterized by new bone formation that progressively leads to ankylosis and functional disability. Due to the limitation of early diagnosis of ankylosing spondylitis (AS), an updated criterion in recent years introduced the concept of axial spondyloarthritis (axSpA) [1] and nonradiographic axial spondyloarthritis (nr-axSpA) [2] and facilitates classification management of disease. The new concept also brings new challenges for the disease. It has been controversial whether the two subphenotypes are separate or different phases of radiographic progression.

The pathogenesis of spondylitis is so far not very clear but has proved to be characterized by inflammation and bone formation. It is still uncertain for the triggering factors of osteophyte induced by chronic inflammation. Studies have revealed that the Wnt/*β*-catenin pathway contributed to the bone fusion in AS [3]. As Wnt signaling inhibitors, Dkk-1 expression was dysfunctional in serum samples from the AS patients [4–9]. The expression of Dickkopf- (DKK-) 1 and sclerostin (SOST) was reduced in the spine of proteoglycan-induced spondylitis (PGISp) mice [10], and blockade of DKK-1 induces fusion of the sacroiliac joints, transgenic for tumour necrosis factor (TNFtg) mice [11], implicating that the Wnt pathway as a likely mediator of the mechanism by which inflammation induces bony ankylosis in SpA.

Myeloid-related proteins (MRP) 8 (S100A8) and14 (S100A9) are endogenous TLR-4 ligands that are expressed in granulocytes and monocytes. The extracellular complex of MRP8 and MRP14 (MRP8/14) is also known as calprotectin. They cause strong proinflammatory effects on phagocytes and endothelial cells in vitro and promote inflammatory processes in vivo. In experimental antigen-induced arthritis, MRP8 and MRP14 significantly contribute to joint inflammation and leucocyte infiltration [12–15]. Calprotectin was reported to be elevated in serum [16] and feces [17] and is an independent marker for radiographic spinal progression in axSpA [18].

Here, we studied the relationship between serum levels of calprotectin in the two subtypes of SpA as well as their correlation with Wnt/*β*-catenin pathway key elements.

## 2. Methods

### 2.1. Study Design and Patient Enrollment

We enrolled 53 patients with AS, 59 patients with nr-axSpA, and 47 healthy individuals. The study was approved by the ethics committee. All participating subjects gave written consent. Patients were diagnosed with AS or nr-axSpA according to the modified New York criteria [19] or ASAS classification criteria for axSpA [1]. Patients with associated inflammatory bowel disease (IBD) were excluded from the analysis.

### 2.2. Outcome Measures

Laboratory tests including ESR, CRP, and human leukocyte antigen- (HLA-) B27 were conducted. Bath Ankylosing Spondylitis Disease Activity Index (BASDAI), Bath Ankylosing Spondylitis Functional Index (BASFI), and Ankylosing Spondylitis Disease Activity Score (ASDAS) were used for the evaluation of disease activity and progression. Imaging assessment was calculated using Spondyloarthritis Research Consortium of Canada (SPARCC) scoring system [20] for the sacroiliac joints and modified Stoke's Ankylosing Spondylitis Spine Score (mSASSS) [21]. Serum levels of calprotectin (Cusabio Biotech Co., China), GSK-*β*, *β*-catenin, and RUNX2 (EIAab Science Co. Ltd, Wuhan, China) were determined by commercial ELISA kit.

### 2.3. Statistical Analysis

Data were described as mean and SD. Mann–Whitney *U* test or Kruskal-Wallis test was used to compare continuous variables. Correlations were assessed using Spearman's rank correlation coefficient. Statistical analyses were performed with SPSS V.13.0 software. A *p* value < 0.05 was considered statistically significant.

## 3. Results

**Figure 1:**
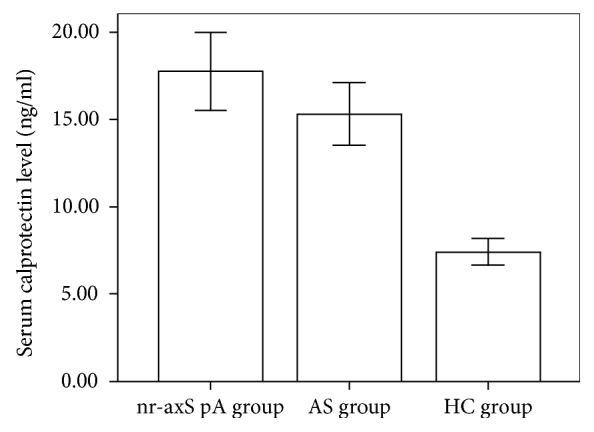
Serum calprotectin level in the nr-axSpA group, AS group, and HC group. Serum calprotectin level (ng/ml) was significantly higher in the AS (14.16 ± 5.32) and nr-axSpA (17.76 ± 8.59) patients than that in the healthy individuals (7.40 ± 2.67) (*p* < 0.05). No difference was observed in calprotectin level between the AS and nr-axSpA patients (*p* > 0.05).

Clinical characteristics and laboratory results in the AS and nr-axSpA patients were shown in Table 1. Age and disease duration (yrs) were 32.3 ± 8.21 and 5.17 ± 3.55 in the AS group and 34.4 ± 7.79 and 4.98 ± 4.14 in the nr-axSpA group, respectively. HLA-B27 positivity was 86.79% (46/53) and 81.36% (48/59) in the AS group and nr-axSpA group, respectively (*p* = 0.455). The male sex rate was 66.04% (male to female: 35/18) and 54.24% (male to female: 32/27) in the AS group and nr-axSpA group, respectively (*p* = 0.248). Serum calprotectin level (ng/ml) was higher in the AS (15.30 ± 6.49) and nr-axSpA (17.76 ± 8.59) patients than that in the healthy individuals (7.40 ± 2.67). No difference was observed in calprotectin level between the AS and nr-axSpA patients (*p* = 0.093) (Figure 1).

**Figure 2:**
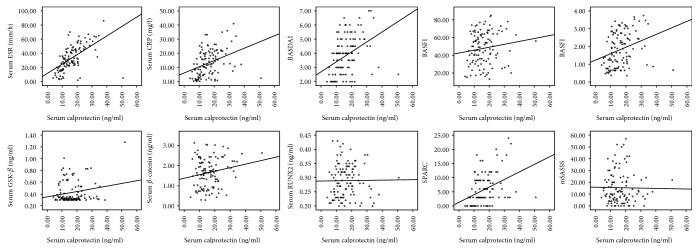
Correlation between serum calprotectin and laboratory results and clinical measurements. Correlation between serum calprotectin and ESR (*r* = 0.679, *p* = 1 × 10^−6^), CRP (*r* = 0.431, *p* = 2.06 × 10^−6^), BASDAI (*r* = 0.481, *p* = 1 × 10^−6^), BASFI (*r* = 0.154, *p* = 0.105), ASDAS (*r* = 0.378, *p* = 3.93 × 10^−5^), GSK-*β* (*r* = 0.034, *p* = 0.722), *β*-catenin (*r* = 0.118, *p* = 0.215), RUNX2 (*r* = 0.092, *p* = 0.336), SPARCC (*r* = 0.405, *p* = 9.21 × 10^−6^), and mSASSS (*r* = −0.033, *p* = 0.726) in patients with nr-axSpA and AS was shown.

Elevated calprotectin was positively correlated with ESR (*r* = 0.679, *p* = 1 × 10^−6^), CRP (*r* = 0.431, *p* = 2.06 × 10^−6^), BASDAI (*r* = 0.481, *p* = 1 × 10^−6^), and ASDAS (*r* = 0.378, *p* = 3.93 × 10^−5^) as well as SPARCC scoring (*r* = 0.405, *p* = 9.21 × 10^−6^) and had no correlation with BASFI (*r* = 0.154, *p* = 0.105) and mSASSS (*r* = −0.033, *p* = 0.726) in these two subgenotypes. No correlation was observed between calprotectin and Wnt/*β*-catenin pathway markers. Radiographic progression indicated by mSASSS was correlated merely with disease duration (*r* = 0.682, *p* = 1 × 10^−6^) instead of other outcome measurements (Figure 2).

## 4. Discussion

There have been contradictory results on the expression of calprotectin in spondylitis. Calprotectin was reported to be elevated in SpA as compared to the healthy controls and decreases rapidly and consistently upon effective treatment [16], while in another work, no differences in serum calprotectin between the AS patients and healthy controls were found [17]. However, previous study has proved that serum calprotectin levels are predictive for the progression of structural damage in the spine in axSpA. Baseline calprotectin serum level was significantly higher in patients with mSASSS worsening versus those without mSASSS worsening, indicating that calprotectin represents a novel predictive biomarker for radiographic spinal progression in axial SpA [18]. If calprotectin facilitates radiographic progression of the disease, is it possible that it might be the trigger for inflammation-induced bone information?

Nr-axSpA and AS (a radiographic form of axial SpA) share common epidemiological, genetic, and clinical characteristics, supporting the concept of axSpA as one entity. About 12% of the patients with nr-axSpA progress to AS over 2 years. Elevated CRP and active sacroiliitis on MRI are the strongest predictors for such a progression [22]. Nr-axSpA seems to be, however, more heterogeneous than AS because of the presence of patients with a self-limiting disease or a slow disease course. The clinical characteristics of the patients with nr-axSpA and AS in our study were in accordance with the previous report. However, differences existed in several aspects. First, female to male ratio was similarly higher in patients with nr-axSpA than in those with AS while the significance was not reached. Second, there was also no significant difference in HLA-B27 positivity between the two groups. Third, no significance was observed for CRP between patients with nr-axSpA and AS. There might be explanations for these discrepancies. Female patients who presented with mild symptoms might not visit the clinic while patients with more severe symptoms were usually with higher disease activity and higher CRP level and were HLA-B27 positive as well.

In our study, calprotectin was identified to be elevated in both the nr-axSpA and AS patients compared to the healthy controls and correlated well with ESR, CRP, BASDAI, ASDAS, and SPARCC indicating bone marrow edema, indicating that it is a valuable marker for inflammation signal. Meanwhile, there is no difference between the nr-axSpA and AS patients in calprotectin levels, which means calprotectin was not able to discriminate these two clinical subtypes. Thus, nr-axSpA might be the early stage of SpA, while it will not necessarily proceed to AS, and calprotectin might not be the intermediary link between the two subtypes.

Previous study demonstrated that extracellular S100A8 and S100A9 proteins contribute to colorectal carcinoma cell survival and migration via Wnt/*β*-catenin pathway as one of the underlying molecular mechanisms of the disease, revealing the possible crosstalk between calprotectin and the Wnt/*β*-catenin pathway [23]. In vitro studies indicated that S100A8/A9 proteins exert their effects via the activation of canonical Wnt signaling. Intra-articular injection of S100A8 increased canonical Wnt signaling, whereas canonical Wnt signaling was decreased after the induction of experimental osteoarthritis in S100A9-deficient mice. S100A8 stimulation of macrophages resulted in increased expression of canonical Wnt signaling members [24]. During the activation of the canonical Wnt signaling, TCF/LEF transcriptional activity was greatly increased by the depletion of DKK1 or GSK3*β*. This allows the accumulation of nuclear *β*-catenin and finally the activation of the transcription of *β*-catenin-dependent genes. Canonical Wnt signaling promotes osteogenesis by directly stimulating RUNX2 gene expression. RUNX2 activates osteocalcin, which is an osteoblast-specific gene expressed by differentiated osteoblasts. Nonetheless, serum calprotectin was not correlated to the Wnt signaling elements in correlation analysis, revealing that calprotectin-related inflammation was not a trigger for subsequent bone infusion caused by aberrant Wnt pathway.

Spondyloarthritis patients can present with extra-articular manifestation such as IBD, and fecal calprotectin was frequently used in the assessment of disease activity, response to treatment, prediction of disease relapse, or postoperative recurrence in IBD [25]. In a longitudinal study of fecal calprotectin in AS, elevated fecal calprotectin at baseline was the strongest predictor of the development of IBD. The fecal calprotectin may be a potential biomarker to identify patients with AS at risk of developing IBD [26]. Since IBD itself might contribute to the elevation of calprotectin level, we have excluded those associated with IBD in the spondyloarthritis patients enrolled in order to avoid confounding effect of IBD.

There are several limitations for our study. The sample size is relatively small to draw more conclusive results. Further study would enroll more individuals to explore confirmative conclusions. Only serum samples were analyzed in our study, whether fecal level of calprotectin was also elevated and the relationship between calprotectin level and intestinal involvement of the patients was not investigated. Besides, toll-like receptor- (TLR-) 4 was the endogenous immunoreceptor of calprotectin, and the combination of the two can activate the TLR4/MyD88 pathway and lead to the secretion of NF-*κ*B and proinflammatory factors including TNF-*α* and IL-17 [27]. The level of members of this pathway was not further evaluated in our study. Long-term follow-up favors further investigation of the value of inflammation and subsequent bone information.

In conclusion, serum calprotectin can be used as a marker for inflammation in both nr-axSpA and AS, while calprotectin-related inflammation was not related with subsequent bone formation of the disease.

## Figures and Tables

**Table 1:** Clinical characteristics and laboratory results in the AS and nr-axSpA patients.

	AS group	nr-axSpA group
Age (mean ± SD, years)	32.3 ± 8.21	34.4 ± 7.79
Disease duration (mean ± SD, years)	5.17 ± 3.55	4.98 ± 4.14
ESR (mm/h)	32.21 ± 16.97	34.58 ± 18.54
CRP (mg/l)	13.75 ± 8.61	13.11 ± 10.16
BASDAI	3.44 ± 1.11	4.20 ± 1.44
BASFI	46.87 ± 17.96	48.78 ± 18.85
ASDAS	1.72 ± 0.97	1.91 ± 0.96
Calprotectin (ng/ml)	15.30 ± 6.49	17.76 ± 8.59
GSK-*β* (ng/ml)	0.32 ± 0.02	0.53 ± 0.21
*β*-catenin (ng/ml)	1.69 ± 0.79	1.63 ± 0.65
RUNX2 (ng/ml)	0.28 ± 0.05	0.30 ± 0.07
SPARCC	5.60 ± 5.24	5.88 ± 5.72
mSASSS	18.57 ± 14.72	12.49 ± 11.48
